# High 4-1BB Expression in PBMCs and Tumor Infiltrating Lymphocytes (TILs) in Patients with Head and Neck Squamous Cell Carcinoma

**DOI:** 10.1055/s-0043-1764419

**Published:** 2023-05-02

**Authors:** Fardeela Bin-Alee, Nattharee Chunthagonesupawit, Tamonwan Meesakul, Areeya Diloktaweewattana, Patnarin Mahattanasakul, Apiwat Mutirangura, Komkrit Ruangritchankul, Somboon Keelawat, Nakarin Kitkumthorn

**Affiliations:** 1 Faculty of Medicine, Princess of Naradhiwas University, Narathiwat, Thailand; 2 Master of Science Program in Medical Sciences, Faculty of Medicine, Chulalongkorn University, Bangkok, Thailand; 3 Division of Dengue Hemorrhagic Fever Research, Faculty of Medicine Siriraj Hospital, Mahidol University, Bangkok, Thailand; 4 Siriraj Center of Research Excellence in Dengue and Emerging Pathogens, Faculty of Medicine Siriraj Hospital, Mahidol University, Bangkok, Thailand; 5 Department of Otolaryngology, Head and Neck Surgery, King Chulalongkorn Memorial Hospital, Thai Red Cross Society, Bangkok, Thailand; 6 Department of Otolaryngology, Head and Neck Surgery, Faculty of Medicine, Chulalongkorn University, Bangkok, Thailand; 7 Center of Excellence in Molecular Genetics of Cancer and Human Diseases, Department of Anatomy, Faculty of Medicine, Chulalongkorn University, Bangkok, Thailand; 8 Department of Pathology, Faculty of Medicine, Chulalongkorn University, Bangkok, Thailand; 9 Department of Oral Biology, Faculty of Dentistry, Mahidol University, Bangkok, Thailand

**Keywords:** 4-1BB, head and neck cancer, PBMCs, TILs

## Abstract

**Objective** 4-1BB is a costimulatory immune-activating molecule. Increased amounts of this protein have previously been found in the plasma of patients with oropharyngeal and oral cancer. Here, we focused on this molecule that functions as part of the immune system. We investigated *4-1BB* in the peripheral blood mononuclear cells (PBMCs) and tumor infiltrating lymphocytes (TILs) of patients with head and neck squamous cell cancer (HNSCC).

**Materials and Methods** The expression level of *4-1BB* in the PBMCs was determined using real-time polymerase chain reaction (PCR). The TIMER (Tumor Immune Estimation Resource) web server was utilized to approximate the *4-1BB* level in HNSCC TILs. Moreover, 4-1BB immunohistochemistry (IHC) was used to validate TILs in four organs of HNSCC, including oral cancer (OC), oropharyngeal cancer (OPC), sinonasal cancer (SNC), and laryngeal cancer (LC), in both the tumor area and adjacent normal epithelium. The difference in 4-1BB expression levels in various groups was assessed using a Kruskal-Wallis test and an independent sample t-test.

**Results** The level of *4-1BB* expression in PBMCs was highest in OPC, followed by OC and healthy controls (HC). Significant differences were discovered between HC and OPC and between OC and OPC. Bioinformatics revealed a substantial correlation between *4-1BB* expression level and lymphocyte infiltration in HNSCC, including B cells, CD8+ T cells, and CD4+ T cells. IHC validation in HNSCC tissue revealed that the average number of 4-1BB positive TILs in all four HNSCC subtypes was considerably greater than the number of lymphocytes seen in adjacent normal tissue. Interestingly, the number of lymphocytes that were 4-1BB positive increased in relation to the TIL level.

**Conclusion** A higher number of *4-1BB* expression levels were found in the PBMCs and TILs of HNSCC patients, implying that 4-1BB may be a promising approach for HNSCC patients to improve their immune function. It is important to study and create a treatment that uses 4-1BB medicine as well as existing drugs.

## Introduction

Head and neck cancer is a cancer that involves the locations of the oral cavity, pharynx, larynx, salivary glands, paranasal sinuses, and nasal cavity. Most head and neck cancers are head and neck squamous cell carcinomas (HNSCCs). 1 This type of cancer develops from the squamous epithelial lining of the surface structures of the head and neck. According to GLOBOCAN, cancers of the oral cavity, larynx, and oropharynx will account for, respectively, 2.5, 0.9, and 0.57% of all cancers in Thailand in 2020. It has also been reported that oral cancer (OC) accounts for 2% of the annual mortality rate, laryngeal cancer (LC) accounts for 8%, and oropharyngeal cancer (OPC) accounts for 4%. 2 There have been numerous efforts made in cancer treatment over the years, including surgery, radiation, chemotherapy, and immunotherapy. Nonetheless, the 5-year survival rate remains disappointing. Consequently, there is still a request for future strategic therapy for this cancer.

4-1BB, which is known as CD137 or TNFRSF9, is a member of the tumor necrosis factor receptor superfamily. 3 4-1BB is found in T cells, B cells, monocytes, and various types of transformed cell lines. This molecule can stimulate T cells, dendritic cells, and monocytes to produce cytokines, proliferate, and mature functionally. 4-1BB binds to the 4-1BB ligand (4-1BBL) to provide T lymphocytes with a co-stimulatory signal. 4-1BB signaling has been linked to antigen presentation and the generation of cytotoxic T lymphocytes (CTL). 3 4 As a result, 4-1BB plays an important role in tumor cell killing by activating CTL. Previous research found that the 4-1BB agonist improved immune cell function and could be used as an immunotherapy. 5 Since studies on the frequency of 4-1BB in each cancer are still scarce, they required information of 4-1BB profiles in tumor infiltrating cells of each tumor.

Based on our earlier findings, secretory 4-1BB levels are higher in the plasma of patients with OC and OPC than in healthy controls (HC). 6 To employ 4-1BB as an immunotherapy, we evaluated the levels of *4-1BB* expression in lymphocytes from HNSCC patients, including peripheral blood mononuclear cells (PBMCs) and tumor infiltrating lymphocytes (TILs). We discovered a greater amount of *4-1BB* in the PBMCs of patients with OC and OPC. Furthermore, using the TIMER (Tumor Immune Estimation Resource) web-based and immunohistochemical techniques, we also found that 4-1BB was more abundant in HNSCC TILs than in adjacent normal tissue.

## Materials and Methods

### Sample Recruitment

All patients with HNSCC were confirmed pathological diagnosis by NK, SK, and KR. This study includes two cohorts as presented in Tables 1 and 2 . In cohort 1, blood samples from 10 HC, 18 patients with OC, and 20 patients with OPC were obtained for 3 mL in an ethylenediaminetetraacetic acid (EDTA) tube. Cohort 2 uses formalin-fixed paraffin-embedded (FFPE) tissue samples from archival specimens from the Department of Pathology, Chulalongkorn University. There was a total of 48 cases of cancer, 12 each of OC, OPC, LC, and sinonasal cancer (SNC). Clinical information about the patient, such as age, gender, and tumor, was obtained from pathologically requested forms or clinical chart record forms. Staging of HNSCC using the 2017 AJCC/UICC staging system. 7 Ethics were reviewed and approved by the Institutional Review Board of the Faculty of Medicine, Chulalongkorn University in Bangkok, Thailand (IRB No. 810/62 and 291/65). Written informed consent was obtained from all patients who participated in this study. Detailed demographic data are demonstrated in Supplementary Tables S1 and S2 (available in the online version).

**Table 1:** Real-time PCR of *4-1BB* expression in PBMCs

PBMCs	*n*	Sex (Male:Female)	Age (y), median (range)	Histological grade (W:M:P)	Clinical stage (1:2:3:4)	Real-time PCR *4-1BB* 2 ^-ΔΔCt^ (avg. ± SD)
Healthy controls (HC)	10	6:4	49.5 (31–65)			1.02 (± 0.22)
Oral cancer (OC)	18	10:8	58.5 (26–76)	11:5:2	4:4:2:8	1.60 (± 0.87)
Oropharyngeal cancer (OPC)	20	16:4	61.5 (35–74)	11:7:2	1:6:2:11	2.73 (± 1.03)

Abbreviations: W, well-differentiated carcinoma; M, moderately differentiated carcinoma; P, poorly differentiated carcinoma; PBMCs, peripheral blood mononuclear cells; PCR, polymerase chain reaction; avg, average; SD, standard deviation.

**Table 2:** Immunohistochemistry percentage of 4-1BB positive TILs in FFPE tissue

FFPE tissue	*n*	Sex (Male:Female)	Age (y), median (range)	Histological grade (W:M:P)	Clinical stage (I:II:III:IV)	%4-1BB positive lymphocyte (avg. ± SD) adjacent normal	%4-1BB positive lymphocyte (avg. ± SD) tumor tissue
Oral cancer (OC)	12	6:6	53 (39–84)	7:5:0	3:3:3:3	13.79 (± 8.12)	6.20 (± 3.63)
Oropharyngeal cancer (OPC)	12	9:3	64 (42–84)	3:7:2	5:3:3:1	13.36 (± 3.52)	4.46 (± 2.06)
Sinonasal caner (SNC)	12	10:2	68.5 (43–80)	5:7:0	5:1:1:5	6.40 (± 3.10)	2.52 (± 1.24)
Laryngeal cancer (LC)	12	12:0	63 (46–80)	8:3:0	1:2:3:7	13.94 (± 9.14)	3.51 (± 1.51)

Abbreviations: FFPE, formalin-fixed paraffin-embedded; W, well-differentiated carcinoma; M, moderately differentiated carcinoma; P, poorly differentiated carcinoma; avg, average; SD, standard deviation; TILs, tumor infiltrating lymphocytes.

### Bioinformatics

To explore the immune cell infiltration of HNSCC microenvironment, bioinformatic data from the TIMER (cistrome.shinyapps.io/timer) were analyzed to determine the expression of *4-1BB* in the immune filtrates of HNSCC. 8 9

On the TIMER online server, we entered specific criteria such as “4-1BB,” “CD137,” and “TNFRSF9,” all of which refer to the same gene, and along with the additional keyword, “HNSCC.” The TIMER results allowed for the examination of various aspects of the tumor, including the tumor's immunological, clinical, and genetic characteristics. In this investigation, we revealed a correlation between the expression of *4-1BB* on individual immune cells and their immunological infiltrates. These immunological infiltrates contained B cells as well as CD4+ and CD8+ T cells.

### Isolation of Peripheral Blood Mononuclear Cells and RNA Extraction

PBMCs were obtained from EDTA blood test through Ficoll–Hypaque density gradient centrifugation, as directed by the manufacturer (GE Healthcare Bio-Sciences AB, Uppsala, Sweden). Afterward, RNA was extracted using the TRIzol LS reagent (Thermo Scientific, Waltham, MA, United States) according to the manufacturer's instructions. The content and purity of RNA were determined using a NanoDrop (ND1000 Spectrophotometer; NanoDrop Technologies, Wilmington, DE, United States).

### Real-Time Polymerase Chain Reaction Analysis

The cDNA was synthesized from RNA by the RevertAid First Strand cDNA Synthesis Kit (Thermo Scientific, Waltham, MA, United States) in accordance with the manufacturer's instructions. The selected candidate gene was amplified with 40 cycles of real-time polymerase chain reaction (PCR). We used the PowerUpTM SYBR Green Master Mix (Thermo Scientific, Waltham, MA, United States) containing the forward primer and the reverse primer of *4-1BB* (5′-GTGTCAAGGCTATTTCAG-3′ and 5′-AGACCACGTCTTTCTCC-3′) with an annealing temperature of 58°C. Glyceraldehyde-3-phosohate dehydrogenase ( *GAPDH* ) was used as an internal control with a forward primer (5′-TGGAAGGACTCATGACCACAG-3′) and a reverse primer (5′-TTCAGCTCAGGGATGACCTT-3′) at 60°C. The expression levels of *4-1BB* and *GAPDH* were measured using the Applied Biosystems QuantStudio 6 Flex Real-Time PCR System (Thermo Scientific, Waltham, MA, United States). All experiments were performed in duplicate and analyzed using the comparative cycle threshold (2 ^−ΔΔCt^ ) approach.

### Immunohistochemistry

Immunohistochemistry (IHC) staining was carried out on FFPE sections, with an appendix portion acting as a positive control. 4-1BB antibody (Cell Signaling Technology, Danvers, Massachusetts, United States) was carried out in IHC for 1 hour and 32 minutes utilizing an automated staining system (Benchmark XT and Ultra; Ventana Medical Systems, Tucson, Arizona, United States). After that, an Aperio CS2 scanner was used to scan the slides (Leica Biosystems, Wetzlar, Germany). The scans were analyzed with Aperio ImageScope version 12.2.2.5015 (Aperio Technologies, Irvine, CA, United States). A total of lymphocytes in the area of the tumor (TILs) and lymphocytes in neighboring normal tissue were counted. In five distinct locations, the studies were carried out by three observers (NC, NK, and KR; *к* less than 0.01). Each area had a total of between 50 and 200 lymphocytes visible (20X magnified). The percentage of 4-1BB positive lymphocytes for each area was calculated as follows: %4-1BB lymphocytes = the amount of the 4-1BB positive lymphocytes divided by the sum of the positive and negative lymphocytes, multiplied by 100 and divided by 5. Finally, the data demonstrated the average percentage of 4-1BB-positive lymphocytes in TILs and adjacent normal lymphocytes areas.

### TILs Evaluation

TILs were counted on the hematoxylin and eosin-stained slide next to the immunologically stained specimen, as the previous study. 10 NC, NK, and KR, three examiners, each separately scored the TILs. Lymphocytes that were seen inside tumor nests or sheets as well as in the stromal layer between tumor nests were included. Neutrophils, plasma cells, and lymphocytes outside of the tumor invasion were not considered while evaluating TILs. The TIL level was evaluated as a semiquantitative parameter and classified as levels 1 (0–25%), 2 (>50%), 3 (>75%), and 4 (>100%) across the entire section.

### Statistical Analysis

The statistical analysis was carried out using SPSS version 22. (SPSS Inc., Chicago, IL, United States). An independent sample *t* -test and Kruskal–Wallis test were performed to determine the difference in *4-1BB* expression levels between and among groups. Fisher's exact test and the chi-squared test were used to determine the association between clinicopathological variables and *4-1BB* expression levels. The association between *4-1BB* expression and immune cell subtypes was calculated using Spearman's correlation coefficient. The *p* -values less than 0.01 were considered statistically significant.

## Results

### *4-1BB* Expression Elevated in the PBMCs of Patients with OC and OPC

**Fig. 1:**
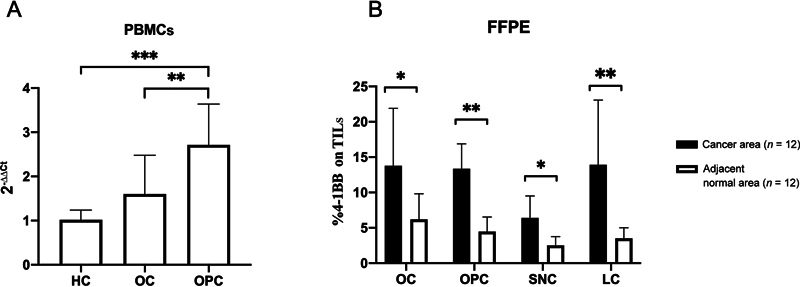
Expression of 4-1BB in peripheral blood mononuclear cells (PBMCs) and tumor infiltrating lymphocytes (TILs) of head and neck squamous cell cancer (HNSCC) patients. Real-time polymerase chain reaction (PCR) of 4-1BB expression in PBMCs. ( **A** ) The levels of 4-1BB expression are increased from healthy controls (HC), oral cancer (OC), and oropharyngeal cancer (OPC). Immunohistochemistry level of 4-1BB positive TILs. ( **B** ) Percentage of 4-1BB positive TILs in four HNSCC types is higher than lymphocytes in the adjacent normal tissues. Denote; *p < 0.01, **p 0.001, and ***p < 0.0001.

*4-1BB* expression in PBMCs was validated by real-time PCR. The results revealed that the 2 ^–∆∆Ct^ values of 4-1BB in HC, OC patients, and OPC patients were 1.02 ± 0.22, 1.60 ± 0.87, and 2.73 ± 1.03, respectively ( Fig. 1A , Table 1 ). These data showed significant differences ( *p* < 0.001) between groups of HC vs OPC and OC vs OPC. The level of *4-1BB* in OC patients is higher than that in HC, but not significantly. We did not find a significant correlation between *4-1BB* expression and sex, age, cancer stage, or histological grade in any of the cancer samples.

### 4-1BB Levels Correlated to the Number of TILs in HNSCC Patients

**Fig. 2:**
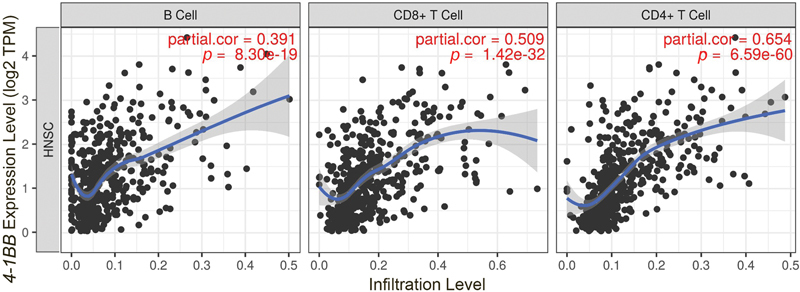
Partial correlation between *4-1BB* expression and immunological infiltrates of ( **A** ) B cell, ( **B** ) CD8 + T cell, and ( **C** ) CD4 + T cell using Tumor Immune Estimation Resource (TIMER).

We used TIMER to investigate the relationship between *4-1BB* expression levels and immune cell infiltration in HNSCC tissue. The TIMER calculates 540 HNSCC tumor tissues and 44 normal head and neck tissues. In Fig. 2 , we focused on TILs and found that *4-1BB* expression had a significant partial correlation with lymphocytes infiltration in HNSCC, including B cells (correlation = 0.391; *p* = 8.30e-19), CD8+ T cells (correlation = 0.509; *p* = 1.42e-32), and CD4+ T cells (correlation = 0.654; *p* = 6.59 e-60; Fig. 2 ). The high level of *4-1BB* expression correlates with increased tumor local lymphocytes infiltration, which is an important prognostic factor in HNSCC patients.

### 4-1BB Expression Elevated in the TILs of Patients with HNSCC

**Fig. 3:**
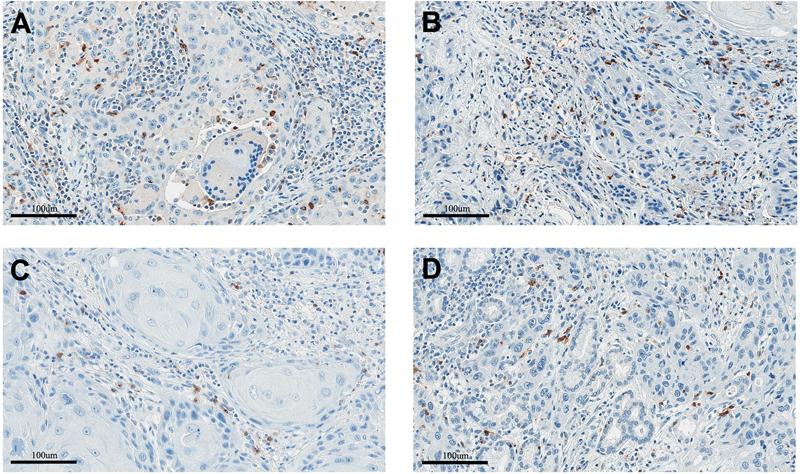
Demonstration of immunohistochemistry (IHC) 4-1BB positive tumor infiltrating lymphocytes (TILs) in ( **A** ) oral cancer (OC), ( **B** ) oropharyngeal cancer (OPC), ( **C** ) sinonasal cancer (SNC), and ( **D** ) laryngeal cancer (LC).

Following that, we looked for 4-1BB positive TILs in HNSCC. We went on to perform IHC on four different types of HNSCC, including OC, OPC, SNC, and LC. Brown staining in the cytoplasm and cell membrane of lymphocytes revealed 4-1BB positive lymphocytes, as shown in ( Fig. 3 ). The presence of 4-1BB on lymphocytes in the tumor region (TILs) was compared to lymphocytes in adjacent normal tissue. We discovered that the percentage of 4-1BB positive lymphocytes observed in TILs was significantly higher than the percentage of lymphocytes observed in neighboring normal tissue in all cases (pair *t* -test; *p* < 0.001; Fig. 1B ). These findings imply that 4-1BB molecules are advantageous in the presence of HNSCC.

**Fig. 4:**
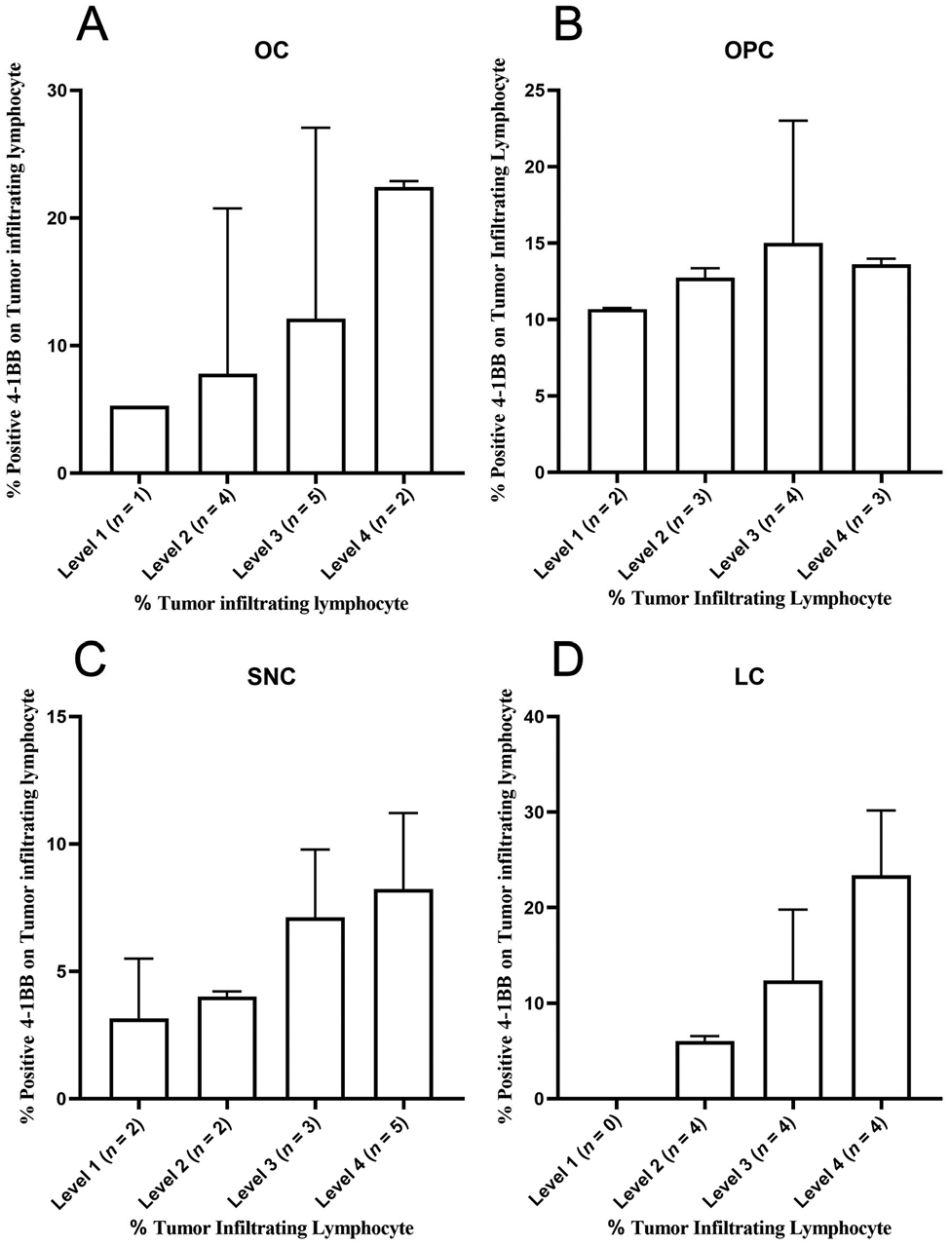
Percentage of 4-1BB positive tumor infiltrating lymphocytes (TILs) in ( **A** ) oral cancer (OC), ( **B** ) oropharyngeal cancer (OPC), ( **C** ) sinonasal cancer (SNC), and ( **D** ) laryngeal cancer (LC). TIL levels are classified as level 1 (0–25%), level 2 (>25–50%), level 3 (>50–75%), and level 4 (>75–100%).

TIL levels were further classified and evaluated based on a 4-1 BB positive cells ratio. The results from the OC and SNC indicated a trend toward an increase in the proportion of 4-1BB positive cells from levels 1 to 4 of TILs ( Fig. 4A, C ). The ratio of 4-1BB positive cells in OPC increased from level 1 to 3, then slightly decreased in level 4 ( Fig. 4B ). While in LC, where we did not have a specimen at level 1, the ratio of 4-1BB positive cells increased from level 2 to 4 ( Fig. 4D ). The overall results of four tumors revealed associations that were comparable to the TIMER results.

## Discussion

Tumor immunotherapy has two signals to regulate T-cell activation, including MHC complexes interacting with T-cell receptors and co-stimulation between T cells and antigen-presenting cells. 3 4 To control the balance in the immune system, co-stimulatory and coinhibitory factors, or immune checkpoint proteins, work together.

4-1BB belongs to the co-stimulatory molecule family. It protects CD8+ T cells against activation-induced cell death. Few studies have examined the amounts of the protein 4-1BB in various cancer cells such as lung, pancreas, etc. 11 12 13 Our study found increased expression of *4-1BB* in PBMCs from OC and OPC compared to HC ( Fig. 1A ). The same findings were observed in *4-1BB* expression levels in TILs of OC, OPC, LC, and SNC ( Fig. 1B ). This is consistent with another study that discovered *OX40, ICOS, GITR,* and *4-1BB* were overexpressed in HNSCC patients' peripheral blood lymphocytes (PBLs) and TILs. 14

Furthermore, we used TIMER to confirm this information. TIMER is just one web service that has been utilized in a comprehensive investigation of individual immune cells that have infiltrated tumors and their expression patterns. 8 9 We also found a partial correlation between the number of TILs in the major lymphocytes and 4-1BB positive lymphocytes ( Fig. 2 ). Taken together, our findings confirmed that 4-1BB is an important activating co-stimulatory molecule found in HNSCC, which should respond to induce cytokine production and promote immune response cells to kill tumor cells. 15 16

Nowadays, treatment of HNSCC is surgical resection and postoperative radiotherapy according to the tumor stage and origin site. 17 18 There is no evidence in clinical trials of SNC-targeted therapy, but there were some effective in vitro experiments of exon 20 mutation SNC by epidermal growth factor receptor gene (EGFR) exon 20 inhibitor targeted therapy, neratinib, which sparked a debate about the viability of SNC treatment. 19 20 On the other hand, the treatments in OC, OPC, and LC have gained potential to use targeted therapy and immunotherapy, such as nivolumab and cetuximab. Nivolumab and cetuximab are monoclonal antibody-based therapies that target Programmed death 1 (PD-1) and EGFR, respectively. 21 22

Immunotherapy using 4-1BB is being developed for a better way of treating HNSCC, in which it is proposed to activate the anticancer immune response cells, B cells, CD4+ T cells, and CD8 + T cells, and suppress the oncogenes. 23 The mechanism of agonistic anti-4-1BB stimulates and activates immune cells by binding with 4-1BB receptors on TILs and increasing proliferation of TILs, resulting in upregulation of an active immune response against the cancer cells. The interaction between 4-1BB and the 4-1BB ligand may restore effector function. For cancer patients, 4-1BB inhibitors should be considered the treatment of choice. One of these are the immunological costimulatory molecule 4-1BB agonists: urelumab and utomilumab.

Previous research has demonstrated that utomilumab and other immunotherapy combination drugs have the ability to act as a 4-1BB agonist in gastric cancer, cervical cancer, melanoma, HNSCC, and non-small-cell lung cancer. It has been demonstrated that urelumab enhances the efficacy of various immunotherapies by enhancing NK-cell survival, DC maturation, and tumor antigen cross-presentation. 24 25 26 27 However, due to medication toxicity, the 4-1BB antibodies must be used as an adjuvant option with other pharmaceuticals or combined treatments for improved performance. 26 28 Anticancer drugs on the market today target immunological checkpoint molecules such as PD-1, cytotoxic T-cell antigen-4 (CTLA-4), and T-cell immunoglobulin mucin-3 (TIM-3). Consequently, combining multiple medications, such as PD-1 with 4-1BB, or Programmed death-ligand 1 (PD-L1) with 4-1BB, to improve the safety and efficacy of cancer therapies is an alternative novel cancer immunotherapy strategy. 4 13 29

## Conclusion

The current study found a higher number of *4-1BB* expression levels in HNSCC patients' PBMCs and TILs, suggesting that 4-1BB may be a promising option for HNSCC patients to boost their immune function. It is critical to research and develop a treatment that incorporates a 4-1BB medication as well as existing medications.

## Funding

This work was supported by the National Science and Technology Development Agency, Thailand (Research Chair Grant, P-19-50189).
