# Supplementary material for: High 4-1BB Expression in PBMCs and Tumor Infiltrating Lymphocytes (TILs) in Patients with Head and Neck Squamous Cell Carcinoma
Source: Eur J Dent. 2023 May 2;18(1):236–42. doi: 10.1055/s-0043-1764419 (PMC10959597; doi:10.1055/s-0043-1764419)
Supplement: Supplementary file 1 — Supplementary Material [file 10-1055-s-0043-1764419-s2312596.pdf]

Supplementary Material Table S1 Detailed data of *4-1BB* real-time polymerase chain reaction results

Code	Sex	Age (y)	Primary organ	Histological grade	Clinical stage	*4-1BB* Real-time PCR ($2^{-\Delta\Delta Ct}$)
HC1	M	53	–	–	–	0.81
HC2	M	31	–	–	–	0.75
HC3	M	45	–	–	–	0.93
HC4	M	65	–	–	–	1.25
HC5	M	26	–	–	–	0.94
HC6	F	48	–	–	–	0.89
HC7	M	57	–	–	–	0.92
HC8	F	30	–	–	–	1.39
HC9	F	60	–	–	–	0.99
HC10	F	51	–	–	–	1.31
OC1	M	70	Buccal mucosa	WD	II	0.85
OC2	F	43	Tongue	MD	II	0.89
OC3	M	50	Tongue	MD	III	0.99
OC4	M	61	Floor of mouth	MD	IV	1.33
OC5	F	26	Floor of mount	MD	I	1.01
OC6	F	60	Gingiva	WD	II	1.55
OC7	F	62	Hard palate	WD	II	0.87
OC8	M	54	Tongue	WD	IV	0.92
OC9	M	71	Gingiva	WD	IV	2.03
OC10	M	57	Buccal mucosa	WD	I	2.39
OC11	F	43	Tongue	WD	IV	1.68
OC12	M	33	Tongue	PD	I	3.58
OC13	M	41	Tongue	WD	IV	1.78
OC14	M	46	Hard palate	WD	IV	3.27
OC15	F	62	Gingiva	WD	I	2.75
OC16	F	76	Gingiva	MD	IV	1.31
OC17	F	61	Gingiva	PD	IV	0.88
OC18	M	68	Tongue	WD	III	0.78
OP1	M	35	Tonsil	PD	IV	4.41
OP2	M	55	Tongue base	WD	IV	2.73
OP3	F	57	Tonsil	MD	II	3.95
OP4	M	62	Tongue base	MD	III	1.19
OP5	M	58	Soft palate	MD	II	2.78
OP6	M	61	Post-pharyngeal wall	WD	IV	3.98
OP7	M	55	Soft palate	MD	II	1.56
OP8	M	56	Tongue base	WD	IV	3.44
OP9	M	70	Tonsil	WD	IV	3.11
OP10	M	43	Soft palate	WD	I	2.97
OP11	M	73	Tongue base	MD	III	3.16
OP12	M	62	Tonsil	WD	IV	3.05
OP13	F	67	Soft palate	WD	IV	1.15
OP14	M	69	Tonsil	WD	IV	4.30

 © 2023. The Author(s).

Supplementary Material Table S1 (*Continued*)

Code	Sex	Age (y)	Primary organ	Histological grade	Clinical stage	*4-1BB* Real-time PCR ($2^{-\Delta\Delta Ct}$)
OP15	M	74	Tongue base	MD	II	3.01
OP16	M	37	Soft palate	PD	IV	2.84
OP17	F	38	Tongue base	WD	IV	1.71
OP18	M	62	Tongue base	WD	IV	0.99
OP19	M	80	Soft palate	WD	II	3.64
OP20	F	64	Tonsil	MD	II	2.31

Abbreviations: MD, moderately differentiated carcinoma; PD, poorly differentiated; WD, well-differentiated carcinoma.

Supplementary Material Table S2 Detailed data of 4-1BB immunohistochemistry results

Code	Sex	Age (y)	Primary organ	Histological grade	Clinical stage	TIL level	%4-1BB positive lymphocyte	
							Tumor area	Adjacent normal area
OC1	F	84	Tongue	WD	I	3	19.91	4.17
OC2	M	51	Tongue	WD	IV	3	12.11	4.17
OC3	F	44	Tongue	MD	IV	2	5.91	4.55
OC4	M	52	Tongue	WD	III	1	5.31	4.76
OC5	F	39	Tongue	MD	III	2	20.75	3.33
OC6	F	64	Tongue	WD	I	3	10.08	8.77
OC7	F	66	Tongue	WD	IV	4	21.98	16.69
OC8	M	50	Tongue	WD	II	4	22.89	4.17
OC9	M	41	Tongue	MD	II	2	5.62	5.81
OC10	M	54	Floor of mouth	MD	I	2	6.08	6.73
OC11	M	65	Tongue	MD	III	3	27.07	4.76
OC12	F	71	Gingiva	WD	II	3	7.78	6.54
OPC1	M	62	Base of tongue	WD	II	4	13.99	5.93
OPC2	M	80	Soft palate	WD	II	1	10.62	2.50
OPC3	M	58	Base of tongue	WD	I	1	10.75	4.50
OPC4	M	68	Soft palate	MD	III	3	14.28	3.56
OPC5	M	42	Base of tongue	MD	II	3	15.75	7.28
OPC6	F	65	Tonsil	PD	I	2	10.19	4.50
OPC7	F	63	Tonsil	PD	IV	2	12.74	2.22
OPC8	M	61	Tonsil	MD	I	4	10.97	1.11
OPC9	M	81	Base of tongue	MD	I	3	23.01	8.06
OPC10	F	52	Tonsil	MD	I	2	13.36	4.86
OPC11	M	77	Soft palate	MD	III	4	13.63	5.71
OPC12	M	84	Tonsil	MD	III	3	11.00	3.33
SNC1	M	53	Sinus	MD	III	4	3.40	1.45
SNC2	M	78	Sinus	MD	IV	3	4.43	1.85
SNC3	M	51	Sinus	MD	IV	1	4.81	2.94
SNC4	M	65	Nasal cavity	WD	IV	4	11.31	3.23

(*Continued*)

 © 2023. The Author(s).

Supplementary Material Table S2 (*Continued*)

Code	Sex	Age (y)	Primary organ	Histological grade	Clinical stage	TIL level	%4-1BB positive lymphocyte	
							Tumor area	Adjacent normal area
SNC5	F	71	Sinus	MD	I	4	9.34	1.15
SNC6	F	72	Sinus	WD	IV	1	1.51	3.15
SNC7	M	66	Nasal cavity	WD	IV	3	9.77	1.92
SNC8	M	80	Nasal cavity	MD	I	4	7.66	1.23
SNC9	M	43	Sinus	MD	I	2	3.88	5.25
SNC10	M	77	Sinus	MD	I	4	9.42	1.52
SNC11	M	61	Sinus	MD	I	2	4.16	3.92
SNC12	M	71	Sinus	MD	II	3	7.14	2.62
LC1	M	46	Larynx	MD	IV	4	26.59	5.90
LC2	M	80	Larynx	MD	IV	4	23.06	3.57
LC3	M	57	Larynx	MD	III	3	21.05	2.78
LC4	M	49	Larynx	WD	IV	4	29.79	3.13
LC5	M	64	Larynx	WD	IV	3	15.21	4.06
LC6	M	65	Larynx	WD	I	2	5.45	3.57
LC7	M	76	Larynx	WD	IV	2	6.67	5.26
LC8	M	62	Larynx	WD	IV	2	6.16	1.79
LC9	M	78	Larynx	WD	IV	3	3.78	1.56
LC10	M	74	Larynx	WD	II	4	14.04	5.72
LC11	M	60	Larynx	WD	III	3	9.45	3.13
LC12	M	61	Larynx	WD	III	2	6.01	1.67

Abbreviations: MD, moderately differentiated carcinoma; TIL, tumor infiltrating lymphocyte; WD, well-differentiated carcinoma; PD, poorly differentiated carcinoma.

 © 2023. The Author(s).
